# Multi-omics analyses of human colorectal cancer revealed three mitochondrial genes potentially associated with poor outcomes of patients

**DOI:** 10.1186/s12967-021-02939-7

**Published:** 2021-06-26

**Authors:** Wei Zhang, Liewen Lin, Ligang Xia, Wanxia Cai, Weier Dai, Chang Zou, Lianghong Yin, Donge Tang, Yong Xu, Yong Dai

**Affiliations:** 1 Department of Clinical Medical Research Center, Guangdong Provincial Engineering Research Center of Autoimmune Disease Precision Medicine, The Second Clinical Medical College, Jinan University (Shenzhen People’s Hospital), Shenzhen, 518020 China; 2 The First Affiliated Hospital, Jinan University, Guangzhou, China; 3 College of Natural Science, University of Texas at Austin, Austin, 78721 USA; 4 Department of Nephrology, Institute of Nephrology and Blood Purification, The First Affiliated Hospital of Jinan University, Jinan University, Guangzhou, 510632 China; 5 The First Affiliated Hospital of Shenzhen University, Shenzhen Second People’s Hospital, Shenzhen, 518028 China

**Keywords:** Prognostic biomarkers, Mitochondria, Multi-omics study, Colorectal cancer, Ribosome

## Abstract

**Background:**

The identification of novel functional biomarkers is essential for recognizing high-risk patients, predicting recurrence, and searching for appropriate treatment. However, no prognostic biomarker has been applied for colorectal cancer (CRC) in the clinic.

**Methods:**

Integrated with transcriptomic data from public databases, multi-omics examinations were conducted to search prognostic biomarkers for CRC. Moreover, the potential biological functions and regulatory mechanism of these predictive genes were also explored.

**Results:**

In this study, we revealed that three mitochondrial genes were associated with the poor prognosis of CRC. Integrated analyses of transcriptome and proteome of CRC patients disclosed numerous down-regulated mitochondrial genes at both mRNA and protein levels, suggesting a vital role of mitochondria in carcinogenesis. Combined with the bioinformatics studies of transcriptomic datasets of 538 CRC patients, three mitochondrial prognostic genes were eventually selected out, including HIGD1A, SUCLG2, and SLC25A24. The expression of HIGD1A exhibited a significant reduction in two subtypes of adenoma and six subtypes of CRC, while the down-regulation of SUCLG2 and SLC25A24 showed more advantages in rectal mucinous adenocarcinoma. Moreover, we unveiled that these three genes had common expressions and might collaboratively participate in the synthesis of ribosomes. Our original multi-omics datasets, including DNA methylation, structural variants, chromatin accessibility, and phosphoproteome, further depicted the altered modifications on their potential transcriptional factors.

**Conclusions:**

In summary, HIGD1A, SUCLG2, and SLC25A24 might serve as predictive biomarkers for CRC. The biological activities they involved in and their upstream regulators we uncovered would provide a functional context for the further-in-depth mechanism study.

**Graphic abstract:**

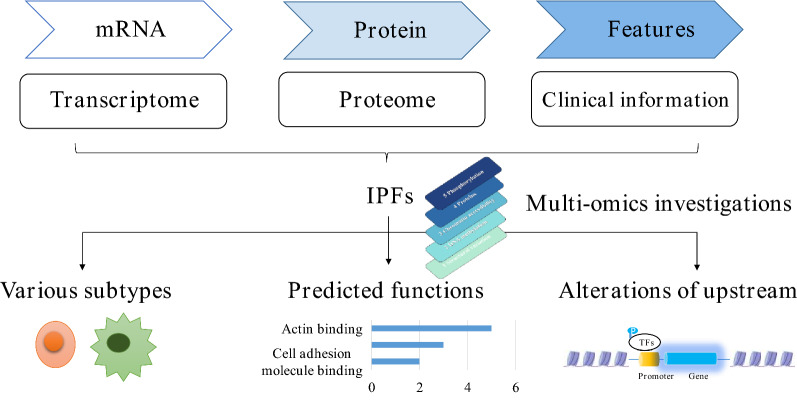

**Supplementary Information:**

The online version contains supplementary material available at 10.1186/s12967-021-02939-7.

## Background

Colorectal cancer (CRC) is the fourth leading cause of all cancer mortality. It kills nearly 900,000 people a year and accounts for 10% of cancer-inducing deaths [1]. CRC has an annual mortality rate of over 40%, despite the recent emergence of promising new therapies, such as targeted- and immune-therapy [2, 3]. One of the most important reasons for the high death rate is that only the early-stage patients could benefit from treatment, but no significant improvement for patients with distant metastases [4]. Therefore, it is necessary to search for effective predictive biomarkers to identify high-risk patients and develop appropriate treatment strategies to improve their prognosis.

Abnormal mitochondrial function is closely related to CRC [5–11]. At the opening stage of CRC, mitochondria show excessive consumption and the loss of mitochondria subsequently drives transformation of tumor [6, 7]. In recent years, some studies have identified several mitochondrial genes as potential prognostic biomarkers. However, most studies lack a large-sample cohort, or rarely compare the genes expression between various subtypes of CRC, or the biomarkers have higher correlations with other tumors [12–16], which means more work still need to be done before clinical application.

In this study, intestinal mucosa from eight CRC patients [four early-stage patients (I–II), two late-stage patients (III–IV), and two late-stage patients with systematic metastasis] were collected and analyzed on multiplex dimensionalities. Integrated with the transcriptome data of 538 samples from the Cancer Genome Mapping Project (TCGA) and our original proteomic data, we searched for independent prognostic factors (IPFs) of CRC, and disclosed three mitochondrial genes for predicting the outcomes of CRC patients. Through co-expression enrichment analyses, the biological processes they involved in were also explored. Furthermore, system examinations, including DNA methylation, structural variants, chromatin accessibility, and phosphoproteome, uncovered their potential upstream regulators’ molecular alterations.

## Methods

### Patients

Eight samples of colorectal adenocarcinoma were collected for investigation. The inclusion criteria were: age ≤ 80 years old, histologically confirmed CRC, no serious major organ dysfunction, and no chemotherapy or radiotherapy. Exclusion criteria were: age ≥ 81 years or older, severe major organ dysfunction, prior chemo- or radiation treatment, or a family history of CRC. In each of the colon cancer tissues studied in this study, two independent pathologists confirmed the morphology of CRC and paracancerous normal colorectal tissue using frozen sections stained with hematoxylin and eosin. All clinical information was obtained from the electronic medical records. Patients participating in this research were voluntary and have been informed and signed informed consent. This project was approved by the ethics committee of Shenzhen People’s Hospital.

Intestinal mucosa from eight CRC patients were collected in a pre-determined method [17]. In brief, tissues within 1 h after surgery were included and collected. The tumorous tissues were taken from the colon segment, and the normal adjacent colonic mucosa within 5 cm of the tumor was gained. The samples were laid in liquid nitrogen for more than 3 h and then stored at − 80 °C.

### Studying cohort from TCGA

RNA-Seq datasets of 51 healthy people and 647 CRC patients were downloaded from TCGA (colonic or rectal intestinal mucosa were examined in the RNA-Seq analyses). Samples with incomplete survival time were excluded. Thus 51 healthy peoples and 538 CRC patients in total were collected for the following study.

### Protein isolation and trypsin digestion

With liquid nitrogen, all the tumorous tissues and the normal adjacent mucosa tissues from the eight CRC patients were ground into cell powder. Mixed with the lysis buffer [8 M urea and 1% protease inhibitor cocktaiIII (Merck Millipore, 156535140)], the mixture was broken at a high-intensity by ultrasound equipment (Scientz), and kept on ice at all times. The debris was then centrifuged at 12,000×*g* at 4 °C for 10 min. Finally, the upper layers of the collection were collected, and the protein concentration was measured using the BCA kit (Beyotime P0011-1) according to the manufacturer’s instructions.

The equivalent amount of protein of each sample was taken for enzymatic hydrolysis, and an appropriate amount of standard protein was added. The volume was adjusted by the lysate, mixed with trichloroacetic acid (TCA), to a final concentration 20%. Subsequently, the protein stayed at 4 °C for 2 h. After centrifuged at 4500×*g* for 5 min, the precipitation was collected and washed with precooled acetone 2–3 times. TEAB was used to dissolve the dried precipitation (final concentration = 200 mM). Dispersed by ultrasound, the mixture was added with trypsin at a ratio of 1:50 (protease: protein, m/m) overnight. Finally, Dithiothreitol (DTT) was put into the solution (final concentration = 5 mM), incubated at 56 °C for 30 min, and then was mixed with Iodoacetamide (IAA) (final concentration = 11 mM), standing at room temperature in the dark for 15 min.

### Liquid chromatography mass spectrometry (LC–MS)/MS analyses for proteome and phosphoproteome investigation

The LC–MS/MS analyses were following a standard protocol [18]. The digested proteins (peptides) were dissolved in liquid chromatography (HPLC) mobile phase A and then separated using NanoElute ULTRA-high performance liquid phase system. Mobile phase A is an aqueous solution containing 0.1% formic acid, then loaded into a reversed-phase analytical column (75-μm i.d., 15-cm length columns for proteomic analyses; 100-μm i.d. and 25-cm length columns for phosphoproteomic analyses). Subsequently, for proteomic analysis, 98% acetonitrile with 0.1% formic acid was added into the solvent gradient, the concentration of which was then increased from 6 to 24% for the first 70 min, and increased from 24 to 35% for 14 min, and to 80% for 3 min, and then maintained at 80% for the last 3 min. For phosphoproteomic analysis, acetonitrile with 0.1% formic acid was added into the gradient, the concentration of which was increased then from 2 to 22% for 50 min, and increased from 22 to 35% for 2 min, and increased to 90% for 3 min, and was then maintained at 90% for the last 5 min.

The peptide segments were separated by an ultra-high performance liquid system and then ionized by the capillary ion source. Then the peptide segments were analyzed by timsTOF Pro mass spectrometry. The ion source voltage was set at 1.6 kV, and TOF was used to detect and analyze the peptide parent ion and its secondary fragments. The scanning range of secondary mass spectrometry was set to 100–1700 m/z. The data acquisition mode used the parallel cumulative Serial fragmentation (PASEF) mode. After a first-stage mass spectrometry acquisition, a secondary spectrogram with the parent ion charge in the range of 0–5 was collected in PASEF mode ten times. The dynamic elimination time of tandem mass spectrometry scanning was set as 30 s to avoid repeated parent ion scanning.

### Database searching, protein annotation, and functional enrichment

Maxquant (V1.6.6.0) was used to analyze the MS/MS data. Using the database Homo_sapiens_9606_SP_(2019)1115 (20,380 sequences), the reverse library was added to calculate the false positive rate (FDR) caused by random matching. In addition, common contamination repositories were also added to the database to eliminate contaminated proteins' influence on the results. Trypsin/P was used for the enzyme digestion, and the missing digit was set as 2. The minimum peptide length was set to 7 amino acid residues, and the maximum number of modified peptides was set as 5. In the first and main search, the mass tolerance of precursor ions was 20 PPM, and fragment ions were 0.02 Da. The fixed modification was the alkylation of cysteine, and the variable modification was oxidation of methionine. Finally, the FDR for protein identification and PSM identification was set at 1%.

GO annotations were performed using the Uniprot-Goa database, supplemented by InterProScan (an algorithm based on protein sequences). For KEGG annotations, the identified proteins were annotated primarily using KAAS V.2.0 (KEGG online service tool). The proteins were then matched to the KEGG pathway using KEGG Mapper V2.5. Finally, subcellular enrichment was carried out using Wolfpsort V.0.2 software.

### The database

Level 3 RNA-Seq datasets (processed and standardized data) of normal and CRC tissues were downloaded from TCGA, and samples with incomplete survival time were excluded. Individual files were merged into matrix files, and gene names were converted from Ensembl IDs to gene symbols via the Ensembl database. The datasets of normal and CRC peoples were extracted from the symbol matrix. The survival analyses (Kaplan–Meier chart) and progression-free survival (PFS) were performed using an R package named “Survival V.” The mRNA expression data of HIGD1A, SUCLG2 and SLC25A24 in 410 patients with CRC were obtained from Oncomine. A list of nuclear-coding mitochondrial genes (NCMGs) was downloaded from MitoCarta [11, 19]. The experimental-tested transcription factors for NCMGs were searched from hTFtarget. The protein–protein interactions (PPI) were analyzed using an online tool GENEMANIA.

### IPFs screening

Univariate and multivariate regression Cox analyses were performed on the mRNA expression datasets to explore IPFs for CRC patients. A P-value of less than 0.05 was regarded as significant.

### Statistical analyses

Student’s t test was used to analyze the significance of differential expression of proteins detected out by proteomic investigations. Fisher's exact test was used to analyze the significance of enrichment analyses. A P-value of less than 0.05 was regarded as significant.

## Results

### The integrated analyses of proteome and transcriptome disclosed three mitochondrial genes as prognostic biomarkers of CRC

**Fig. 1:**
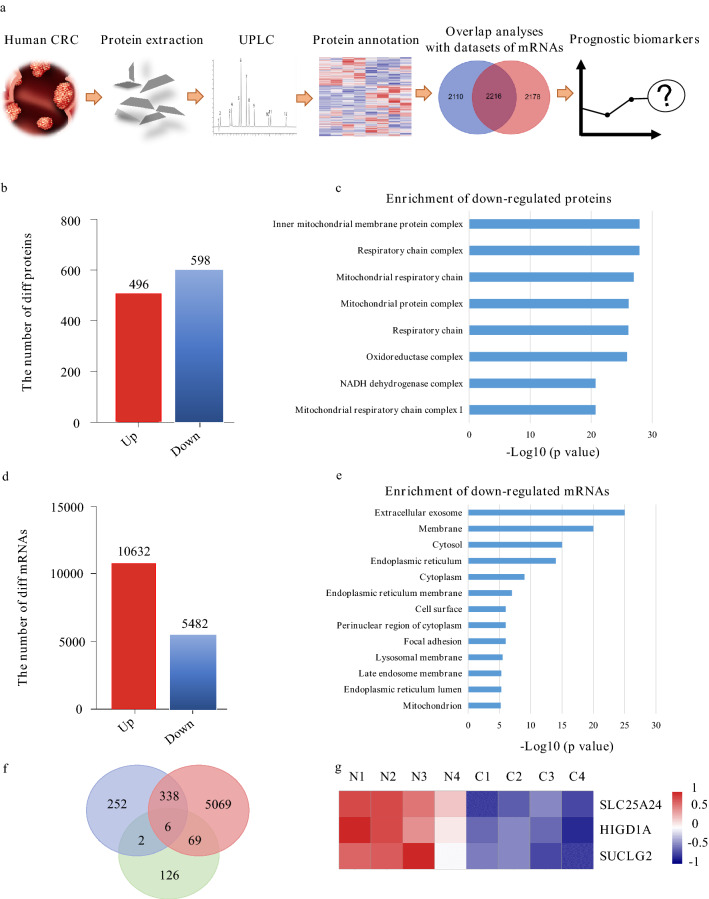
The transcriptome-proteome investigations revealed three mitochondrial genes as potentially prognostic biomarkers for human CRC. **a** Schematic overview of the research. **b** The numbers of differentially expressed proteins in CRC. **c** GO enrichment of the down-regulated proteins. **d** The numbers of differentially-expressed mRNAs in CRC. Genes with a fold change > 1.5, p < 0.05 were regarded as the differentially-expressed genes. **e** GO enrichment of the down-regulated mRNAs. **f** Six IPFs were down-regulated at both mRNA and protein level. **g** SLC25A24, HIGD1A and SUCLG2 were screened out as IPFs for CRC

Since proteins are the basic working units for the human body, we first conducted proteomics examinations on eight CRC patients (four stage I–II patients, two stage III–IV patients, and two stage III–IV patients with systemic metastasis) (Table 1, Fig. 1a). Samples from two patients of each stage were pooled and then performed. As a result, 5451 proteins were quantifiable, in which 598 genes were down-regulated and 496 genes were up-regulated in CRC tissues versus healthy tissues (Fig. 1b). Through GO enrichment of the down-regulated proteins, a large number of mitochondrial proteins were found (Fig. 1c), indicating that mitochondria played an essential role in the development of CRC. And then, we investigated if these expression changes in CRC tissues versus normal adjacent tissues were also occurring at the transcriptional level. Transcriptomic datasets of 538 CRC patients were downloaded from the TCGA (Table 2), and the differentially expressed genes were analyzed. As a result, 10,632 genes were up-regulated, and 5482 genes were down-regulated in CRC tissues versus normal adjacent tissues (Fig. 1d). Through the enrichment analyses of down-regulated genes, the mitochondrial genes were also shown significant expression changes (Fig. 1e). The above results indicated less transcription of mitochondrial genes and a sharp reduction of the mitochondrial proteome. Consistent with previous researches, the inactivity of mitochondria in the opening stage of CRC participates in cellular transformation [6, 7]. Next, we analyzed the transcriptome dataset of 538 CRC patients from TCGA. As a result, 203 IPFs were discovered using univariate and multivariate regression Cox analyses. (Additional file 1: Table S1). After overlapping the 5482 down-regulated mRNA in CRC, and 598 down-regulated proteins in CRC, and the IPFs, we discovered that the expression of six IPFs were both changed at the mRNA and protein levels (Fig. 1f), in which three candidates were mitochondrial genes, including HIGD1A, SUCLG2, and SLC25A24 (Fig. 1g, Additional file 2).

**Table 1:** The characteristics of eight CRC patients in our study

	I–II	III–IV
Age		
≤ 65	0 (0.0%)	1 (12.5%)
> 65	4 (50.0%)	3 (37.5%)
Type		
Adenocarcinoma	4 (50.0%)	4 (50.0%)
Sex		
Male	1 (12.5%)	3 (37.5%)
Female	3 (37.5%)	1 (12.5%)
T-stage		
T1	1 (12.5%)	0 (0.0%)
T2	0 (0.0%)	0 (0.0%)
T3	2 (25.0%)	1 (12.5%)
T4	1 (12.5%)	3 (37.5%)
N-stage		
N0	4 (50.0%)	0 (0.0%)
N1	0 (0.0%)	4 (50.0%)
N2	0 (0.0%)	0 (0.0%)
M-stage		
M0	4 (50.0%)	1 (12.5%)
M1	0 (0.0%)	3 (37.5%)
Location		
Ascending colon	2 (25.0%)	1 (12.5%)
Sigmoid colon	1 (12.5%)	3 (37.5%)
Appendix colon	1 (12.5%)	0 (0.0%)
Chemotherapy		
Yes	0 (0.0%)	0 (0.0%)
No	4 (50.0%)	4 (50.0%)

**Table 2:** The characteristics of 538 CRC patients from TCGA

	Type	Patients
Fustat	Alive	431 (80.1%)
	Dead	107 (19.9%)
Age	≤ 65	232 (43.1%)
	> 65	306 (56.9%)
Type	Adenocarcinoma	538 (98.5%)
	Not clear	8 (1.5%)
Sex	Male	254 (47.2%)
	Female	284 (52.8%)
Stage	I	95 (17.7%)
	II	208 (38.7%)
	III	149 (27.6%)
	IV	86 (16.0%)
T-stage	T1	17 (3.2%)
	T2	92 (17.1%)
	T3	372 (69.1%)
	T4	57 (10.6%)
M-stage	M0	452 (84.0%)
	M1	86 (16.0%)
N-stage	N0	313 (58.2%)
	N1	128 (23.8%)
	N2	97 (18.0%)
Location	Colon	389 (72.3%)
	Rectum	141 (26.2%)
	Not clear	8 (1.5%)

**Fig. 2:**
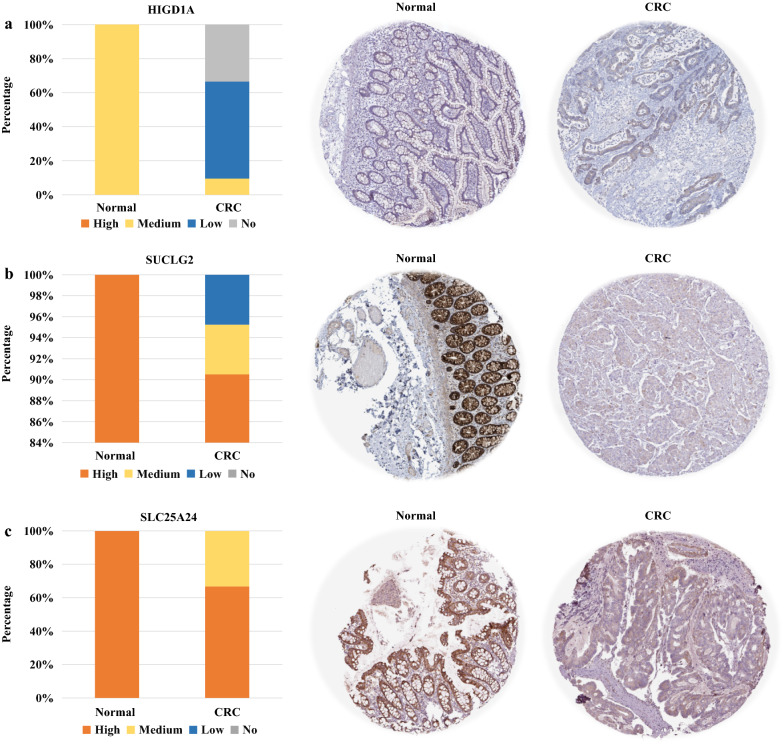
The protein levels of HIGD1A, SUCLG2 and SLC25A24 were decreased in CRC. The immunohistochemical measurement of **a** HIGD1A (normal = 3, tumor = 21), **b** SUCLG2 (normal = 6, tumor = 45) and **c** SLC25A24 (normal = 3, tumor = 21) in the normal and colon adenocarcinoma tissues. The immunohistochemical results were obtained from The Human Protein Atlas. *Normal* normal adjacent tissues, and *CRC* CRC tissues

To further confirm the protein expression of the three genes in human CRC, we also mined their protein expression in The Human Protein Atlas. We found that the expression of HIGD1A was significantly down-regulated or absent in most CRC patients, and SUCLG2 and SLC25A24 were also down-regulated in partial patients (Fig. 2a–c).

**Fig. 3:**
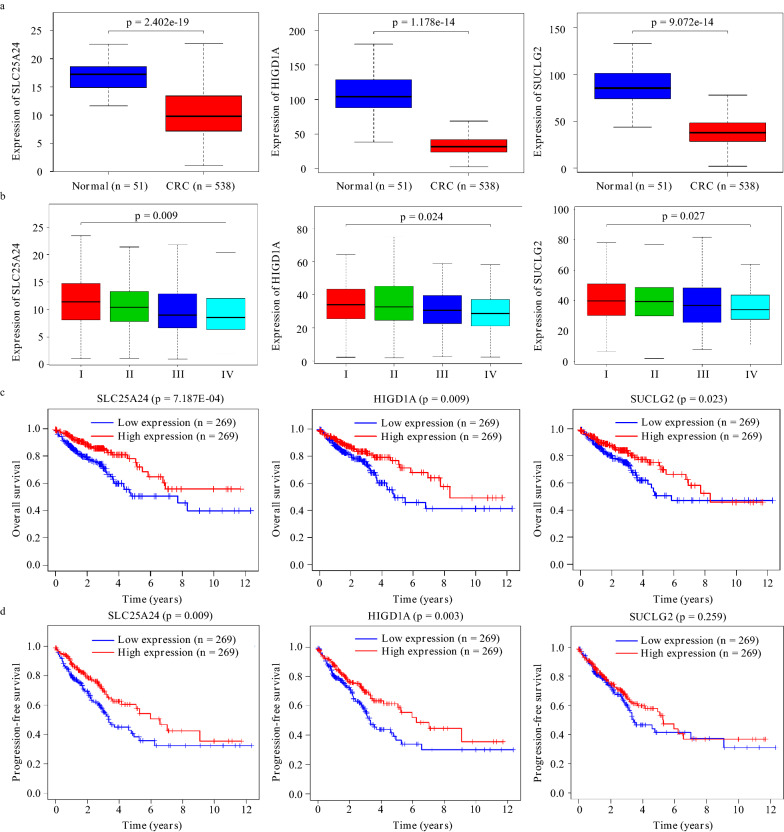
HIGD1A, SUCLG2, and SLC25A24 were associated with the survival of CRC patients. **a** The mRNA expression of the three genes in CRC, and **b** was continuously reduced with the disease progression and negatively correlated to **c** the overall survival rates of the patients and **d** the progression of CRC

For a detailed study, the mRNA expression of HIGD1A, SUCLG2, and SLC25A24 was also analyzed in 538 CRC patients and healthy people (n = 51) from TCGA. Consequently, HIGD1A, SUCLG2, and SLC25A24 were all down-regulated in CRC (Fig. 3a) and exhibited a stably declined expression throughout the tumor progression (Fig. 3b). Furthermore, the relationship between their expression and the overall survival rate (OS) of patients was also investigated with a follow-up threshold of 12 years. After grouped by the median value of expression of the target gene, the OS of patients was significantly declined in low-expression groups (Fig. 3c). Meanwhile, we analyzed the OS in colon and rectal cancer, respectively. The results showed that all three genes were significantly associated with the OS of colon cancer, but only SUCLG2 was associated with the OS of rectal cancer (Additional file 2). Furthermore, the PFS analyses revealed that the decreased expression of HIGD1A and SLC25A24 accelerated the tumor malignancy (Fig. 3d).

**Fig. 4:**
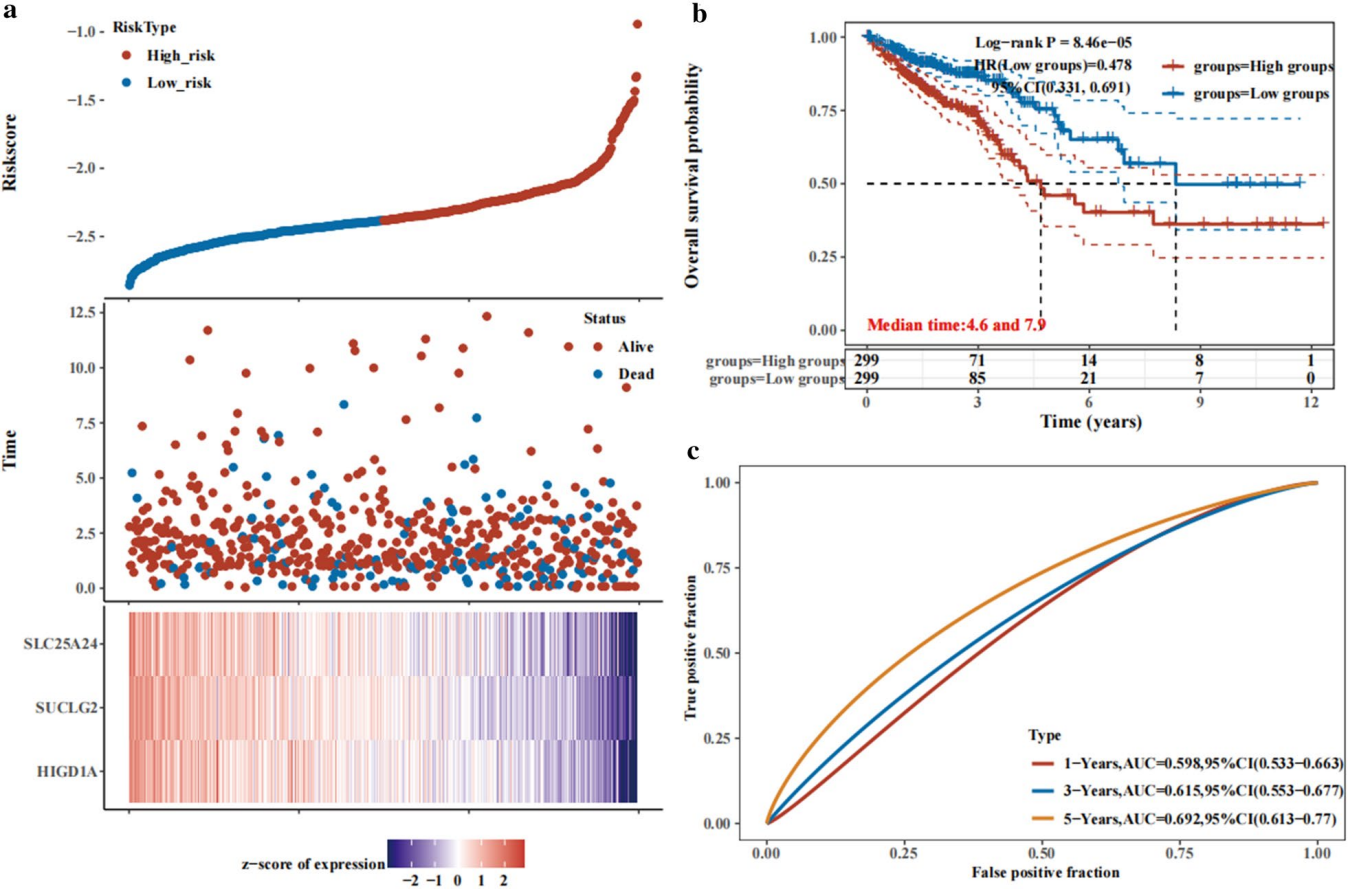
HIGD1A, SUCLG2, and SLC25A24 acted as a signature could differentiate CRC patients at high risk from low risk. **a** CRC patients were divided into high- and low-risk groups according to the expression of three genes. **b** The overall survival rate of patients in high- and low-risk groups. **c** The AUC curve showed effect of the signature for classification

Subsequently, to examine the predictive effect of HIGD1A, SUCLG2, and SLC25A24 on CRC survival when they act as signature, we grouped patients into a high-risk group and low-risk group by the median value of expression of the three genes. The results showed that patients with low expression of the three genes were at high risks (Fig. 4a), and there was a significant difference in the OS between the high-risk group and the low-risk group (Fig. 4b), and the AreaUnderRoc (AUC) curve also showed that the signature has a good value in classification (Fig. 4c).

### The expression of the three genes could complement and verify each other in various subtypes of colorectal tumors

**Fig. 5:**
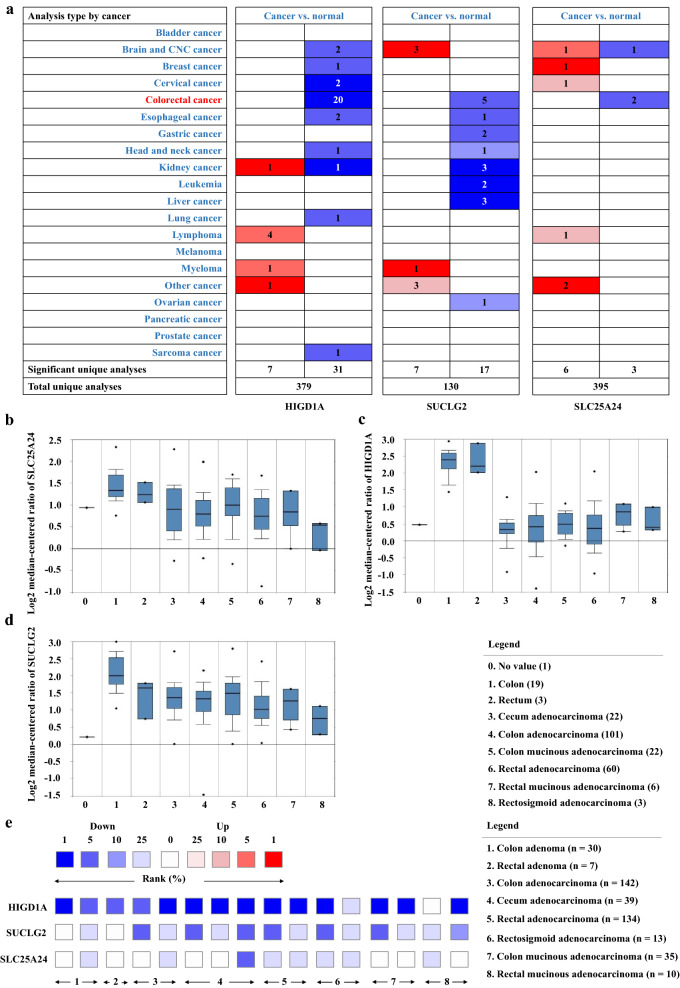
HIGD1A, SUCLG2, and SLC25A24 could complement each other’s detection results in various subtypes of benign and malignant colorectal tumors. The mRNA expression of **a** SLC25A24, HIGD1A and SUCLG2 in unique CRC datasets of Oncomine, and **b** SLC25A24, **c** HIGD1A, and **d** SUCLG2 in different subtypes of colorectal tumors. **e** The expression rank of HIGD1A, SUCLG2, and SLC25A24 in various subtypes of colorectal tumors. The colors represent the gene-rank percentile for the investigations within the cell

Next, to further understand the potentials of HIGD1A, SUCLG2, and SLC25A24 as the IPFs for CRC, we investigated their mRNA expression in unique datasets of CRC and different subtypes of colorectal tumors through the Oncomine database. As a result, the three genes were all down-expressed in 20, five and two independent CRC studies (Fig. 5a). In one independent study, HIGD1A, SUCLG2, and SLC25A24 were all significantly reduced in various subtypes of colorectal tumors, including cecum adenocarcinoma (n = 22), colon adenocarcinoma (n = 101), colon mucinous adenocarcinoma (n = 22), rectal adenocarcinoma (n = 60), rectal mucinous adenocarcinoma (n = 6), and rectosigmoid adenocarcinoma (n = 3) (Fig. 5b–d). Furthermore, another investigation uncovered that the expression of HIGD1A was significantly reduced in two subtypes of adenoma and six subtypes of CRC, but the difference in rectal mucinous adenocarcinoma was significant only in one analysis. On the other hand, SUCLG2 and SLC25A24 were not significantly down-regulated as HIGD1A in adenoma and five subtypes of CRC, while SUCLG2 exhibited consistently decreased expression in both two independent studies of rectal mucinous adenocarcinoma, and SLC25A24 also showed down-regulation in one out of two independent studies (Fig. 5e). This suggested that SUCLG2 and SLC25A24 might be used as helper genes for HIGD1A in clinical diagnosis, which might improve the diagnostic accuracy of rectal mucinous adenocarcinoma.

### Co-expression analyses demonstrated that the three genes might collaboratively participate in the biogenesis of ribosomes or protein translation

**Fig. 6:**
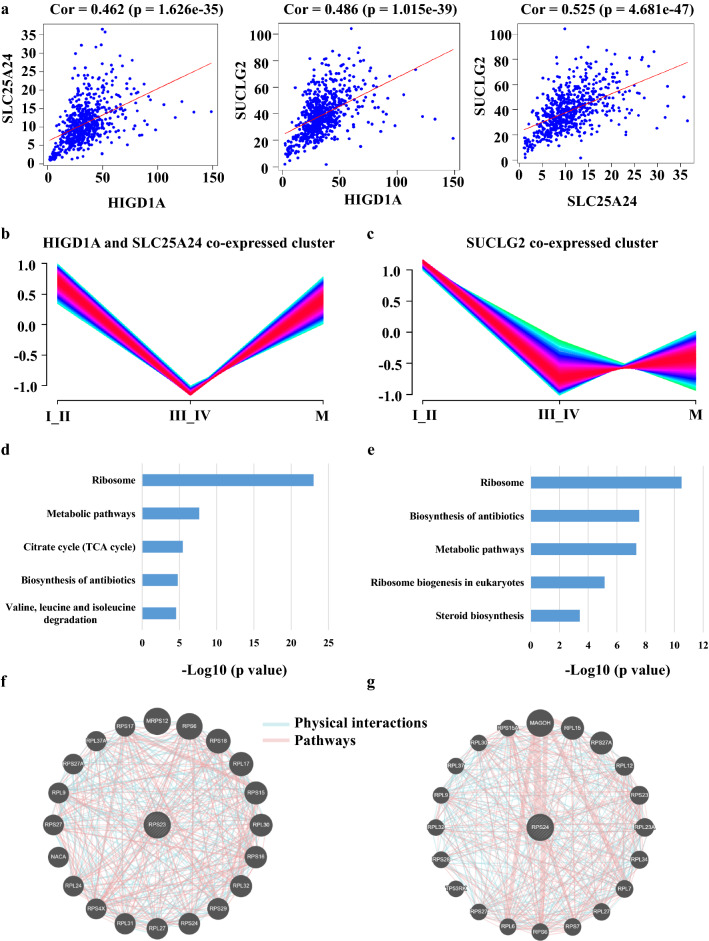
HIGD1A, SUCLG2, and SLC25A24 potentially participated in the ribosome’s biogenesis in a collaborative manner. **a** The mRNAs of HIGD1A, SUCLG2, and SLC25A24 were likely to be co-expressed with each other in human CRC. **b** A cluster of proteins co-expressed with HIGD1A and SLC25A24, and **c** a cluster of proteins co-expressed with SUCLG2. KEGG analyses of **d** HIGD1A and SLC25A24 co-expressed proteins, and **e** SUCLG2 co-expressed proteins. Protein–protein interaction networks of **f** HIGD1A and SLC25A24 co-expressed ribosomal proteins, and **g** SUCLG2 co-expressed ribosomal proteins

As we have known, the three selected-out genes all locate in mitochondria. HIGD1A is identified as a regulator of cytochrome c oxidase and modulates respiratory functions [20, 21]. SUCLG2 is a subunit of succinyl-CoA synthetase, involving the tricarboxylic acid cycle (TCA) [22, 23]. SLC25A24 plays as a carrier protein for the transport of ATP-Mg for phosphate exchanges [24, 25]. Whereas, how they functioned in CRC is not clear. Therefore, we analyzed the co-expressed genes of the three genes basing on the transcriptome datasets of CRC patients. The results showed that the three genes HIGD1A, SUCLG2, and SLC25A24 were co-expressed with each other (Fig. 6a), indicating that they might simultaneously participate in the same biological process or have synergistic effects. Next, we classified all the differentially-expressed proteins based on expression pattern of proteomics detected by the proteomic examinations. We found that HIGD1A and SLC25A24 belong to the same cluster (Fig. 6b), and there were 552 proteins co-expressed with the two genes (Additional file 1: Table S1). SUCLG2 belongs to another cluster, and 741 proteins were expressed together with SUCLG2 (Fig. 6c). Subsequently, we performed KEGG analyses of these co-expressed proteins and observed that a large number of ribosomal proteins were enriched (Fig. 6d, e). The enriched ribosomal proteins, ulteriorly, include cellular and mitochondrial ribosomal proteins. This suggested that the down-regulation of the expression of these three genes might affect the biogenesis of cellular and mitochondrial ribosome. Nevertheless, the ribosome-related proteins co-expressed with HIGD1A and SLC25A24 were utterly different from those co-expressed with SUCLG2. This implied that there might be complementary functions between HIGD1A, SLC25A24, and SUCLG2. These results indicated that the three genes were likely to regulate the ribosomes' biosynthesis collaboratively. Furthermore, we explored the protein–protein interaction of the enriched ribosomal proteins. As a result, most of these proteins have physical interactions, and the potentially central proteins were RPS23 and RPS24 (Fig. 6f, g).

**Fig. 7:**
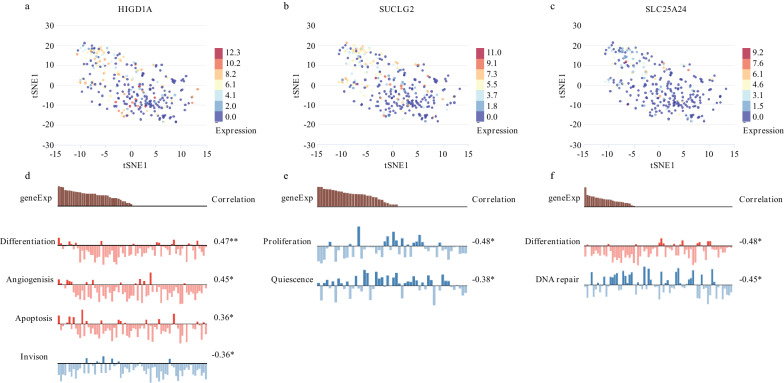
HIGD1A, SUCLG2 and SLC25A24 were potentially associated with a series of tumor-related biological processes such as invasion, angiogenesis and apoptosis. **a**–**c** Difference expression of HIGD1A, SUCLG2 and SLC25A24 was shown in unique CRC cells; **d**–**f** HIGD1A, SUCLG2 and SLC25A24 were potentially related to multi-functions of cells. **p < 0.01, *p < 0.05

Subsequently, we analyzed the tumor-related biological functions the three genes potentially involved in using single-cell-sequencing datasets from CancerSEA. As a result, expression of the three genes exhibited differences in unique CRC cells (Fig. 7a–c). Noticeably, HIGD1A was negatively correlated with invasion, and positively correlated with differentiation, angiogenesis and apoptosis. SUCLG2 was negatively correlated with proliferation and quiescence. SLC25A24 was positively correlated with cell differentiation and negatively correlated with DNA repair (Fig. 7d–f). Because the expression of these three genes is reduced in CRCs, it is likely that they were potential suppressors in CRC, which related to tumor initiation and progression.

### Changes in phosphorylation and chromatin accessibility of the upstream transcriptional factors revealed the underlying mechanisms for the down-regulation of these three genes

**Fig. 8:**
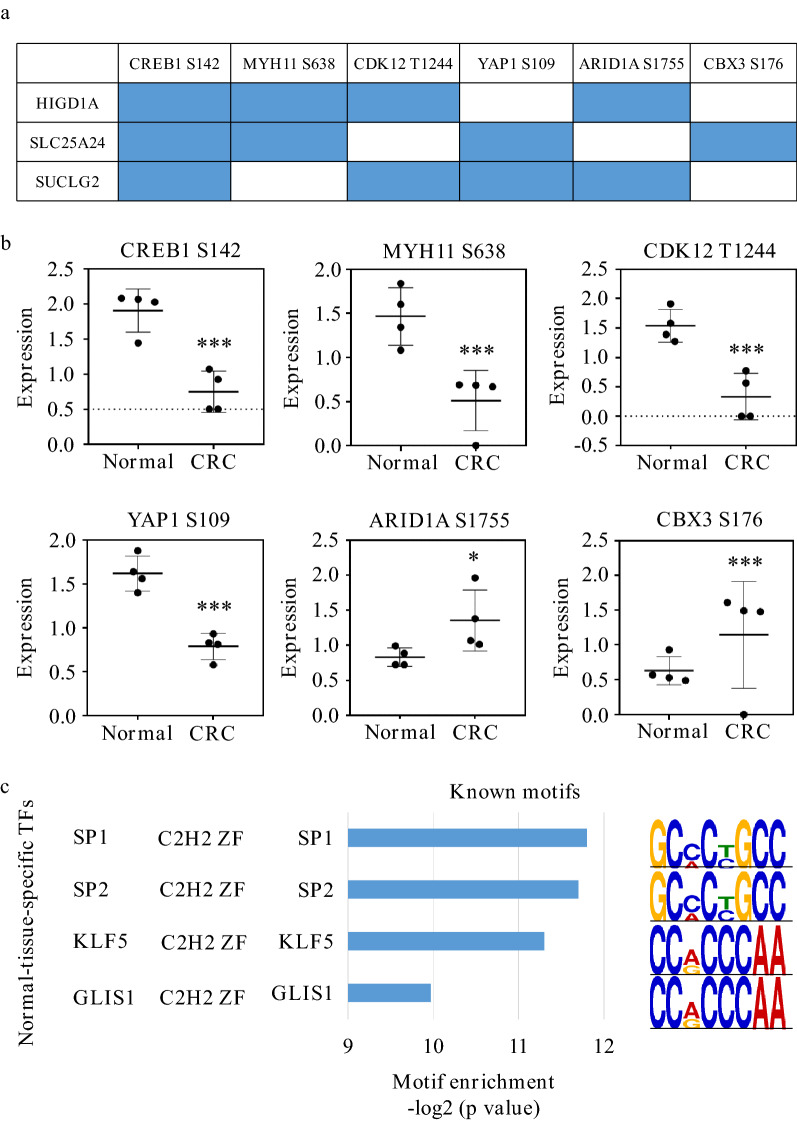
Comprehensive investigations unveiled altered amount of phosphorylation modifications or binding motifs on the three mitochondrial genes' potential transcriptional factors. **a** The regulatory functions of transcription factors CREB1, ARID1A, CDK12, YAP1, MYH11, and CBX3 on HIGD1A, SUCLG2, and SLC25A24. Blue color represents a regulatory relationship. **b** Differentially expressed phosphorylation of the potential transcriptional factors. **c** The specific binding motifs in normal tissues and their corresponding transcriptional factors. ***p value < 0.001, **p value < 0.01, and *p value < 0.05

Next, to investigate the reasons for the reduction of the three genes, six to eight CRC samples were examined on multiplex dimensionalities, including DNA methylation, structural variation, chromatin accessibility and phosphorproteome. We knew that transcription factors played a crucial role in the expression of downstream gene, so we downloaded all transcriptional factors of the three genes that were experimentally verified in the colon from the TFtarget, and investigated their molecular changes using our multiplatform datasets. As a result, six transcriptional factors, showed phosphorylation changes, including CREB1, ARID1A, CDK12, YAP1, MYH11, and CBX3 (Fig. 8a, b). Moreover, we found that the common transcriptional factors of these three genes, SP1, SP2, KLF5, and GLIS1, lacked DNA binding sites in CRC through analyzing chromatin accessibility (Fig. 8c). The results revealed that the changes of phosphorylation on transcription factors or the absence of binding motifs of the transcription factors on DNA might be reasons for the decreased expression of the three genes.

## Discussion

**Fig. 9:**
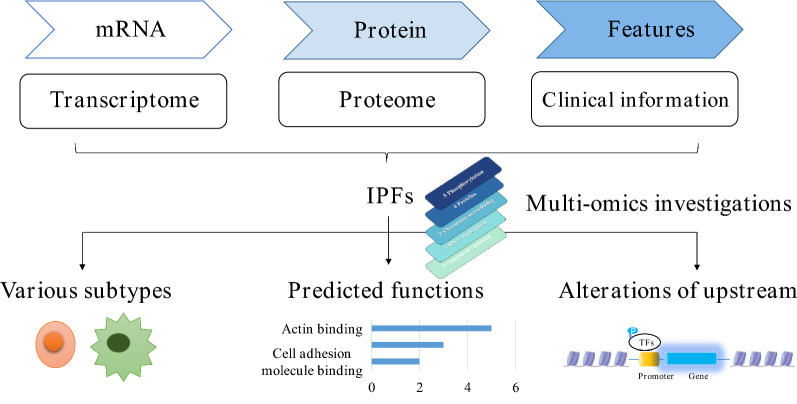
Integrated analysesof human CRC uncovered three mitochondrial genes potentially associated with patients’ outcomes. Through the multiple platform investigations of the three genes, we demonstrated their expression in various subtypes of colorectal tumors, predicted biological processes they involved in, and disclosed the molecular changes of their potential transcriptional factors. *TFs* transcriptional factors

In this study, we found that the mitochondrial proteome was significantly reduced in CRC. Subsequently, we screened out three mitochondrial IPFs for CRC, including HIGD1A, SUCLG2 and SLC25A24. We verified the expression of these three genes in two colorectal adenomas and six CRC subtypes. Furthermore, the enrichment analyses of these three genes’ co-expressed proteins revealed that their role in the regulation of ribosomal synthesis. Finally, multi-omics studies uncovered different phosphorylation and open chromatin of their potential transcriptional factors, which elucidated the possible mechanism of their decreased expression (Fig. 9).

Reliable prognostic biomarkers may help providing more effective treatments for patients at high risk. However, because patients with different clinical characteristics have great heterogeneity, and the identification of prognostic markers requires long-term follow-up observation of patients, which costs a lot of manpower and financial resources, so far, no prognostic marker genes have been applied in the diagnosis of CRC in clinical practice. In CRC progression, 50% of patients face metastasis and recurrence [26], highlighting the importance of discovering of prognostic markers. In this study, we found three mitochondrial genes as potential CRC prognostic biomarkers, which provided new opportunities for clinical diagnosis and treatment.

Meanwhile, mitochondrial dysfunction plays an important role in the development of CRC. In recent years, mitochondrial targeting drugs have attracted much more attention. Some drugs have been approved by the Food and Drug Administration (FDA), including ABT-199 (A BCL2-specific agent), etc. [27]. However, there is still a lack of targeted agents available in the treatment of CRC. Through the analysis of CRC tissues versus normal tissues by omics combined with public database, a large number of CRC related genes have been obtained with high throughput, so that we may have a deeper understanding of the pathogenesis of CRC and obtain evidence for looking for new drug targets.

Protein is the basic unit of biological processes in cells. Through proteomic testing of eight CRC samples, we found a reduction of mitochondrial proteins. This is consistent with previous reports of reduced mitochondrial activity at the early CRC [6, 7]. In tumorigenesis, mitochondrial changes start from cell transformation and continue throughout all stages of CRC. The use of mitochondrial genes as prognostic factors is likely to apply to CRC patients at all phases, from the early stage to the late stage, to the stage of metastasis. We know that the amounts of mRNA and protein of a gene are associated with many factors, including mRNA modification [28, 29], translation efficiency [30, 31], and protein stability and degradation [32, 33]. A change in the expression of a gene on mRNA may not result in a difference in the protein level that would affect the body's physiological activities. In other words, changes in both mRNA and protein levels suggest that the gene may have a more substantial effect on the body than those expressed only at the mRNA level. Simultaneously, if the gene is used as a prognostic marker, the mRNA and protein levels can be detected together for mutual verification. In this study, the protein levels of HIGD1A, SUCLG2, and SLC25A24 screened by us were decreased in CRC patients. Meanwhile, transcriptomic analyses of 538 patients showed that HIGD1A, SUCLG2, and SLC25A24 were also significantly down-regulated in mRNA levels, indicating that these three genes may play a more critical role in the occurrence, development, and prognosis of CRC. Moreover, since all three genes were altered in mRNA and protein levels, future clinical applications can be detected at the mRNA or protein level or both, which makes them more promising as prognostic genes.

SUCLG2, HIGD1A, and SLC25A24, three outcome-associated genes of CRC are functional proteins in mitochondria. They take participate in the biological processes in mitochondria including TCA, cell apoptosis and anaerobic environment [20–25]. SUCLG2 encodes a succinyl-CoA synthetic enzyme, participating in the formation of succinic acid which is an intermediate product of TCA, and is also one of the fermentation products of anaerobic metabolism [22, 23]. The function of HIGD1A is a positive regulator of cytochrome C oxidase and can induce apoptosis under hypoxia. It is associated with hypoxia microenvironment of cells, and when hypoxia-induced factors 1 (HIF-1) lacks, expression of HIGD1A will increases, cooperated with electron transport proteins reducing oxygen consumption and ROS production [20, 21]. SLC25A24 is a carrier protein that exchanges ATP-Mg for phosphate [24, 25].

We found that the three genes might have synergistic expression, and they were positively correlated with the generation or function of the ribosome and mitochondrial ribosome; that is, the reduction of the proteins of the three genes may lead to the decrease in ribosome biogenesis, the weakening of activity, and the decline or slowing down of protein translation. Ribosomogenesis is the most complex and energy-consuming biological process in the body and plays a close role in tumorigenesis [34–39]. Therefore, it can be speculated that, at least at the CRC cell transformation stage, one of the consequences of reduced mitochondrial function may be to induce a reduction in ribosome production, thereby reducing the energy consumption of tumor cells. But whether there is any interaction or synergy between the two biological processes remains unknown. On the other hand, the transcriptome and proteome analyses discovered that more than 16,000 mRNAs but only 1097 proteins were differentially expressed in CRC. This also may be related to the slow translation in CRC cells.

## Conclusions

In summary, the present study found that reduced expression of three mitochondrial genes was associated with poor outcomes (overall survival rates, etc.) of CRC patients, and revealed their potential synergistic effect in the modulation of ribosomes. The findings serve candidate genes for the prognosis of CRC and provide a deeper understanding of the pathogenesis.

## Supplementary Information

**Additional file 1: Table S1.** Down-regulated proteins in CRC patients; down-regulated mRNAs in CRC patients; independent prognostic factors for CRC; co-expressed proteins of HIGD1A, SUCLG2 and SLC25A24; ribosome proteins co-expressed with HIGD1A, SUCLG2 and SLC25A24; experimentally-tested transcriptional factors of HIGD1A, SUCLG2 and SLC25A24 in human colon.

**Additional file 2.** The cox analyses of HIGD1A, SUCLG2 and SLC25A24; The overall survival rates of HIGD1A, SUCLG2 and SLC25A24 in colon and rectum cancer.

## Data Availability

The mass spectrometry proteomics and phosphoproteomics data have been unloaded and deposited onto the ProteomeXchange Consortium through the PRIDE partner repository: PXD021314, PXD021318.

## Abbreviations

CRC: Colorectal cancer
IPFs: Independent prognostic factors
TCGA: The Cancer Genome Atlas Program
NCMGs: The nuclear-coding mitochondrial genes
OS: Overall survival rate
GO: Gene Ontology
KEGG: Kyoto Encyclopedia of Genes and Genomes
PFS: Progression-free survival analyses
PPI: Protein–protein interactions
TFs: Transcriptional factors
PASEF: Parallel accumulation serial fragmentation
FDR: False positive rate
