# Supplementary material for: Multi-omics analyses of human colorectal cancer revealed three mitochondrial genes potentially associated with poor outcomes of patients
Source: J Transl Med. 2021 Jun 26;19:273. doi: 10.1186/s12967-021-02939-7 (PMC8236205; doi:10.1186/s12967-021-02939-7)
Supplement: Supplementary file 2 — Additional file 2. The cox analyses of HIGD1A, SUCLG2 and SLC25A24; The overall survival rates of HIGD1A, SUCLG2 and SLC25A24 in colon and rectum cancer. [file 12967_2021_2939_MOESM2_ESM.docx]

**Title:** Multi-omics analyses of human colorectal cancer revealed three mitochondrial genes potentially associated with poor outcomes of patients

**Running title**: Integrated analyses uncovered novel prognostic biomarkers for CRC

**Authors and affiliations**: Wei Zhang^1，2^, Liewen Lin^1^, Ligang Xia^1^, Wanxia Cai^1^, Weier Dai^3^, Chang Zou^1^, Lianghong Yin^4^, Donge Tang^1^**^*^**, Yong Xu^5^**^*^**, Yong Dai^1^**^*^**

**Author details**

^1^ Department of Clinical Medical Research Center, Guangdong Provincial Engineering Research Center of Autoimmune Disease Precision Medicine, The Second Clinical Medical College, Jinan University (Shenzhen People's Hospital), Shenzhen 518020, China.

^2^ The First Affiliated Hospital, Jinan University, Guangzhou.

^3^ College of Natural Science, University of Texas at Austin, Austin 78721, United States of America.

^4^ Department of Nephrology, Institute of Nephrology and Blood Purification, the First Affiliated Hospital of Jinan University, Jinan University, Guangzhou 510632, China.

^5^ The First Affiliated Hospital of Shenzhen University, Shenzhen Second People's Hospital, Shenzhen 518028, China.

***Corresponding authors**: Donge Tang, Department of Clinical Medical Research Center, Guangdong Provincial Engineering Research Center of Autoimmune Disease Precision Medicine, The Second Clinical Medical College, Jinan University (Shenzhen People's Hospital), Shenzhen 518020, China. E-mail address: [donge66@126.com](mailto:donge66@126.com); Yong Xu, The First Affiliated Hospital of Shenzhen University, Shenzhen Second People's Hospital, Shenzhen 518028, China. E-mail address: [xuyong_2000@tom.com](mailto:xuyong_2000@tom.com); or Yong Dai, Department of Clinical Medical Research Center, Guangdong Provincial Engineering Research Center of Autoimmune Disease Precision Medicine, The Second Clinical Medical College, Jinan University (Shenzhen People's Hospital), Shenzhen 518020, China. Tel/Fax: (86) 0755-22942780. E-mail address: [daiyong22@aliyun.com](mailto:daiyong22@aliyun.com) or dai.yong@szhospital.com.

***Co-Authors:** These authors contributed equally to this work.

**Additional Tables and Figures**

**Table S2 HIGD1A as an independent prognostic gene for colorectal cancer (CRC)**

|  | **Univariate Cox analysis** | | | | **Multivariate Cox analysis** | | | |
| --- | --- | --- | --- | --- | --- | --- | --- | --- |
| **Term** | **HR** | **lower .95** | **upper .95** | **p** | **HR** | **lower .95** | **upper .95** | **p** |
| **age** | 1.0407 | 1.0216 | 1.0601 | 0.0000 | 1.0516 | 1.0321 | 1.0715 | 0.0000 |
| **gender** | 0.9937 | 0.6791 | 1.4541 | 0.9742 | 0.8713 | 0.5896 | 1.2875 | 0.4892 |
| **stage** | 2.2261 | 1.7908 | 2.7673 | 0.0000 | 1.3859 | 0.7474 | 2.5698 | 0.3003 |
| **T** | 2.8826 | 1.9687 | 4.2209 | 0.0000 | 1.8785 | 1.2112 | 2.9134 | 0.0049 |
| **M** | 4.2497 | 2.8558 | 6.3240 | 0.0000 | 1.7888 | 0.7681 | 4.1657 | 0.1776 |
| **N** | 2.0330 | 1.6247 | 2.5439 | 0.0000 | 1.2739 | 0.8668 | 1.8721 | 0.2179 |
| **HIGD1A** | 0.9785 | 0.9651 | 0.9920 | 0.0020 | 0.9812 | 0.9674 | 0.9952 | 0.0086 |

**Table S3 SLC25A24 as an independent prognostic gene for CRC**

|  | **Univariate Cox analysis** | | | | **Multivariate Cox analysis** | | | |
| --- | --- | --- | --- | --- | --- | --- | --- | --- |
| **Term** | **HR** | **lower .95** | **upper .95** | **p** | **HR** | **lower .95** | **upper .95** | **p** |
| **age** | 1.0407 | 1.0216 | 1.0601 | 0.0000 | 1.0492 | 1.0299 | 1.0688 | 0.0000 |
| **gender** | 0.9937 | 0.6791 | 1.4541 | 0.9742 | 0.8207 | 0.5567 | 1.2097 | 0.3181 |
| **stage** | 2.2261 | 1.7908 | 2.7673 | 0.0000 | 1.3128 | 0.6989 | 2.4659 | 0.3975 |
| **T** | 2.8826 | 1.9687 | 4.2209 | 0.0000 | 1.9785 | 1.2675 | 3.0884 | 0.0027 |
| **M** | 4.2497 | 2.8558 | 6.3240 | 0.0000 | 1.9827 | 0.8390 | 4.6857 | 0.1188 |
| **N** | 2.0330 | 1.6247 | 2.5439 | 0.0000 | 1.2539 | 0.8457 | 1.8590 | 0.2602 |
| **SLC25A24** | 0.9351 | 0.8955 | 0.9765 | 0.0024 | 0.9536 | 0.9133 | 0.9956 | 0.0306 |

**Table S4 SUCLG2 as an independent prognostic gene for CRC**

|  | **Univariate Cox analysis** | | | | **Multivariate Cox analysis** | | | |
| --- | --- | --- | --- | --- | --- | --- | --- | --- |
| **Term** | **HR** | **lower .95** | **upper .95** | **p** | **HR** | **lower .95** | **upper .95** | **p** |
| **age** | 1.0407 | 1.0216 | 1.0601 | 0.0000 | 1.0492 | 1.0299 | 1.0690 | 0.0000 |
| **gender** | 0.9937 | 0.6791 | 1.4541 | 0.9742 | 0.8375 | 0.5685 | 1.2338 | 0.3696 |
| **stage** | 2.2261 | 1.7908 | 2.7673 | 0.0000 | 1.3837 | 0.7472 | 2.5622 | 0.3016 |
| **T** | 2.8826 | 1.9687 | 4.2209 | 0.0000 | 1.8809 | 1.2173 | 2.9063 | 0.0044 |
| **M** | 4.2497 | 2.8558 | 6.3240 | 0.0000 | 1.8176 | 0.7807 | 4.2321 | 0.1658 |
| **N** | 2.0330 | 1.6247 | 2.5439 | 0.0000 | 1.2542 | 0.8485 | 1.8539 | 0.2560 |
| **SUCLG2** | 0.9815 | 0.9690 | 0.9940 | 0.0040 | 0.9873 | 0.9745 | 1.0001 | 0.0486 |

**Figure S1 The expression of HIGD1A, SLC25A24 and SUCLG2 was associated with overall survival rate (OS) of colonic cancer and rectal cancer.** In colonic cancer, the OS was associated with the expression of (a) HIGD1A, (b) SLC25A24, (c) SUCLG2. In rectal cancer, the association between OS and the expression of (d) HIGD1A, (e) SLC25A24, (f) SUCLG2.
